# A bibliography of smart nanomaterials biological application in myocardial infarction research

**DOI:** 10.1097/MD.0000000000037672

**Published:** 2024-04-05

**Authors:** Yi Chen, Jianna Zhang

**Affiliations:** a Department of Emergency Medicine, West China Hospital, Sichuan University/West China School of Nursing, Sichuan University, Institute of Disaster Medicine, Sichuan University, Nursing Key Laboratory of Sichuan Province, Chengdu, China.

**Keywords:** bibliography, biological application, myocardial infarction, nanomaterials

## Abstract

Myocardial infarction has been considered the top cause of mortality globally. Numerous studies investigated the biological application of smart nanomaterials in myocardial infarction. Our study aimed to provide an overview of this area through bibliography research. Literature related to the biological application of nanomaterials was retrieved from the web of science core collection database. Bibliography analysis was performed using Microsoft Excel, VOSviewer, Citespace, and the R package “bibliometrix.” A total of 1226 publications were included. The USA, China, and India carried out the most of studies. Harvard University is the most productive institution. Matthias Nahrendorf ranked first in article volume and also owned the highest impact. Keyword burst analysis indicated the frontiers and hotspots to be gold nanoparticles and iron oxide nanoparticles. This bibliography analysis provides a comprehensive overview of uncovered current research trends and emerging hotspots of nanomaterials’ biological application in myocardial infarction, thus inspiring further investigations.

## 1. Introduction

Ischemic heart disease is the leading cause of death globally, with about 9 million deaths annually.^[1]^ It can present as an emergency acute myocardial infarction (MI) where heart muscle dysfunction is caused by atherosclerotic plaque in a coronary artery rupturing resulting in thrombotic occlusion and insufficient blood flow to the myocardium.^[2]^ The common treatment for MI is an immediate restoration of coronary blood flow to reduce the ischemic injury to the myocardium through percutaneous coronary intervention.^[3]^ However, patients still experience significant mortality and morbidity.^[4]^ There is still a need for new treatments to protect the heart from MI’s detrimental effects and to improve the diagnosis, treatment, and overall outcome of MI.

The incorporation of nanomaterials in biological systems has revolutionized the field of biomedical research.^[5–7]^ Smart nanomaterials have emerged as a powerful tool to support the diagnosis and treatment of several diseases, including cancer,^[8,9]^ Alzheimer disease,^[10]^ and COVID-19.^[11]^ Numerous studies have assessed the potential of smart nanomaterials in MI research, including the development of new diagnostic and therapeutic approaches.^[12,13]^ Advanced imaging techniques based on smart nanomaterials have been used to identify the extent of tissue damage induced by MI and to monitor the progression of the disease.^[13]^ Furthermore, innovative nanomaterials have been designed to improve how drugs are delivered to the heart, leading to more effective and targeted therapies.^[14]^ Additionally, the integration of smart nanomaterials in restoring the tissue homeostasis has shown promising results for the repair and regeneration of myocardial extracellular matrix.^[15]^

Integration of scientific output about a certain topic helps scholars master the latest process in such field and the urgent issues to address in future studies. Given the broad range of applications and the benefits of smart nanomaterials in MI, we conducted a bibliography analysis. Different from traditional reviews, the bibliography is based on a quantitative investigation of a wide range of publication information extracted from the database, which could be more objective and comprehensive. Additionally, the bibliography reveals information on publication trends, research group cooperation, and hotspots over time, thus offering a reference guide for researchers and clinicians interested in the potential impact of smart nanomaterials in the field of myocardial infarction research. We also highlight the potential research directions in the future, which can lead to innovative and effective therapeutic strategies for MI patients.

## 2. Materials and methods

### 2.1. Search strategy and data collection

We conducted a literature search related to the biological application of nano-materials on the web of science core collection database on April 20, 2023 (see File S1, Supplemental Digital Content, http://links.lww.com/MD/M27, which detailed the search term). All literature was exported in TXT format of “full records and references,” including title, author, country, published journals, institution, etc. The literature information was also exported into Microsoft Excel 2021 and prepared for subsequent analysis. Information such as the annual publication/citation volume was extracted directly from the citation report provided by web of science.

### 2.2. Scientometric analysis

The annual trends of publication volume were presented by bar and line chart in Microsoft Excel 2021 and the top 10 most-cited references were highlighted in descending order of citation frequency.

With CiteSpace 6.1.R6 Basic, co-authorship of institutions, burst analysis of keywords, and the timeline view of the occurrence of keywords was identified. After removing duplicate research, we set the time cutoff point of analysis as 1 year. Pathfinder and pruning sliced networks were created to highlight the connection diagram of important nodes, which were selected to represent country, institution, or keywords. The color of the node indicates its corresponding cluster.

By VOSviewer v1.6.19, the co-authorship of countries, co-authorship of authors, co-citation analysis of References/Journals/Authors, and occurrence of keywords were conducted. In visual maps, the size of the node is proportional to the value. The connection link between different nodes indicates the co-authorship or co-citation relationship.

The R package “bibliometrix” (R version 4.2.2) was applied for the visualization of country scientific production, the three-filed plot (country, author, and source), and the top 10 authors and journals with the highest impact. The h-index representing academic performance was used to define the impact of individual authors and journals.

## 3. Results

### 3.1. Study inclusion

**Figure 1.**
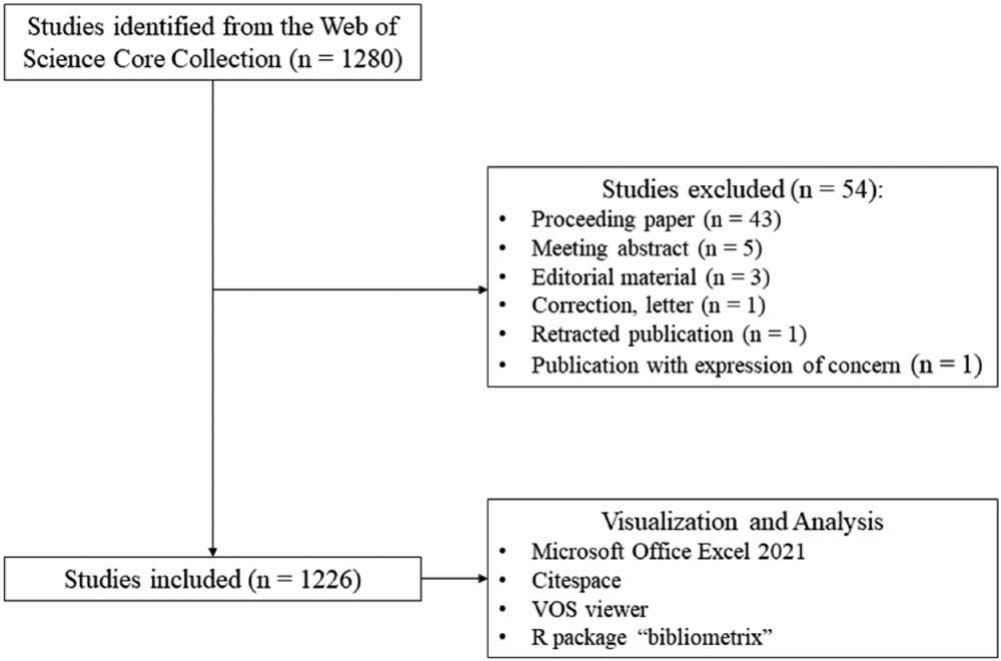
The retrieval flowchart.

Through the search in web of science core collection, a total of 1280 relevant literature were identified. We excluded 54 papers based on the publication types including proceeding papers, meeting abstracts, early access, editorial material, book chapters, corrections, letters, publications with the expression of concern, and retracted publications. Eventually, 1226 articles and review articles were included. The detailed process of selection is shown in Figure 1.

### 3.2. Annual publication/citation variation

**Figure 2.**
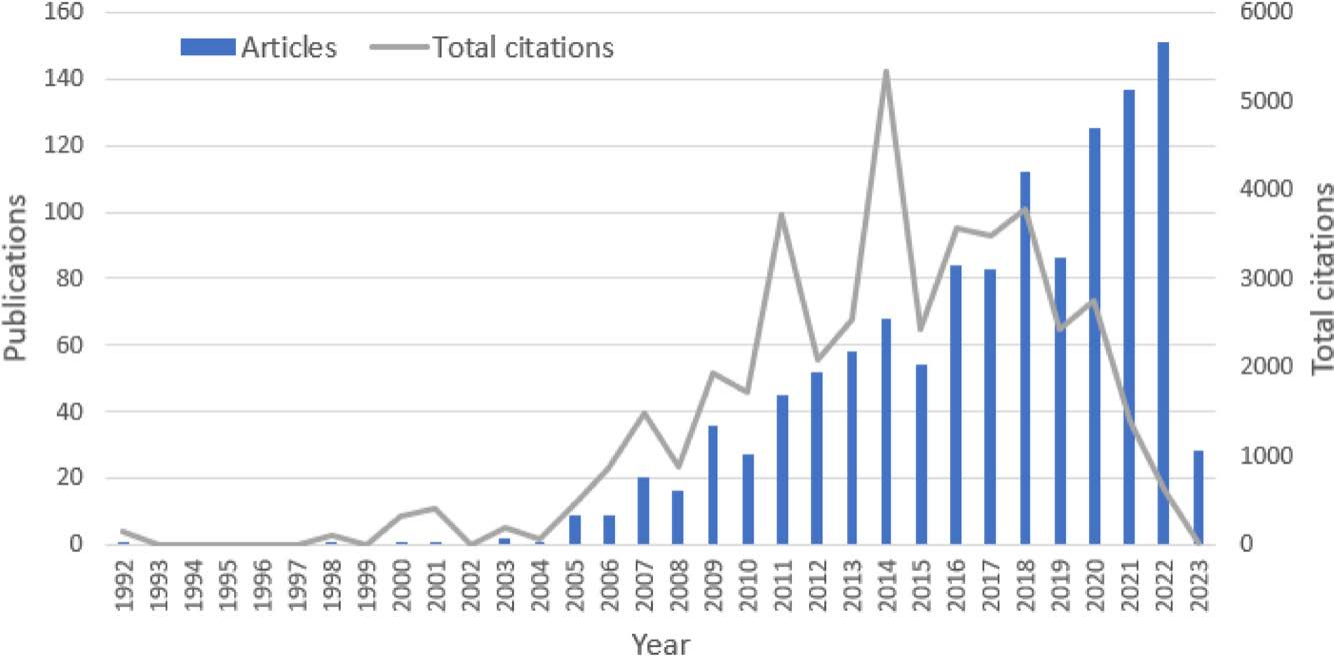
Annual publication and citation number of smart nanomaterials biological application in myocardial infarction research.

As shown in Figure 2, the number of publications and total citations started in 1992 and has risen since about 2004 to 2005 as there were 1 and 9 publications in 2004 and 2005, respectively. Both number of publications and total citations soared in 2011 (45 total publications and 3721 citations) and 2012 (52 total publications and 2089 citations). The trends diverged from then on. The number of publications increased gradually while the number of total citations experienced 2 steep up-and-downs in 2010 and 2013 and decreased slowly since 2018. In 2022, the number of publications and citations are 151 and 616, respectively.

### 3.3. Contribution analysis of countries/institutions/authors

The contributions of countries (publication volume, citations, and co-authorship), institutions (publication volume and co-authorship), and authors (publication volume and co-authorship) were analyzed. The top 10 active countries/institutions/authors are listed in Table 1.

**Table 1:** The top 10 active countries/institutions/authors contributing to the area of smart nanomaterials biological application in myocardial infarction (sorted by number of publications).

Contributions of the country						
Rank	Country	Records	Citations		Publication start year	h-index
			TC	AAC		
1	USA	384	15,470	51.70	1992	100
2	China	336	8514	25.50	2003	72
3	India	92	1108	15.60	2006	24
4	South Korea	76	2116	37.10	2009	34
5	Iran	71	1240	23.00	2012	28
6	Germany	57	860	28.70	2001	36
7	UK	52	1847	57.70	2000	35
8	Italy	48	496	18.40	2009	29
9	Japan	42	1425	40.70	2001	35
10	Canada	41	923	36.90	2004	40
Contributions of the institution						
Rank	Institution	Records	Citations		Publication start year	h-index
			TC	AAC		
1	Harvard University	61	5463	89.55	1992	50
2	Harvard Medical School	47	4564	97.10	1992	38
3	Chinese Academy of Sciences	36	1486	41.28	2010	24
4	Massachusetts General Hospital	35	3393	96.93	1992	30
5	University of California System	25	1583	63.33	2003	41
6	Indian Institute of Technology System (IIT System)	20	317	15.84	2014	11
7	Universidade de São Paulo	20	266	13.30	2013	6
8	University of Texas System	19	1232	64.86	2014	6
9	Stanford University	19	1089	57.29	2012	24
10	Kyushu University	19	1047	55.10	2009	21
Contributions of the author						
Rank	Author	Records	Citations		Publication start year	h-index
			TC	AAC		
1	Nahrendorf, Matthias	23	3393	147.52	2005	22
2	Egashira, Kensuke	18	992	55.11	2009	18
3	Matoba, Tetsuya	18	992	55.11	2009	18
4	Weissleder, Ralph	17	3233	190.18	1992	20
5	Nakano, Kaku	14	899	64.21	2009	15
6	Maranhao, Raul C	11	345	31.36	2007	12
7	Anderson, Daniel G	8	1445	180.60	2009	9
8	Gopinath, Subash C B	8	235	29.38	2015	5
9	Iwamoto, Yoshiko	8	1434	179.25	2011	9
10	Koga, Jun-ichiro	8	518	64.75	2009	10

AAC = average article citation, TC = total citation.

**Figure 3.**
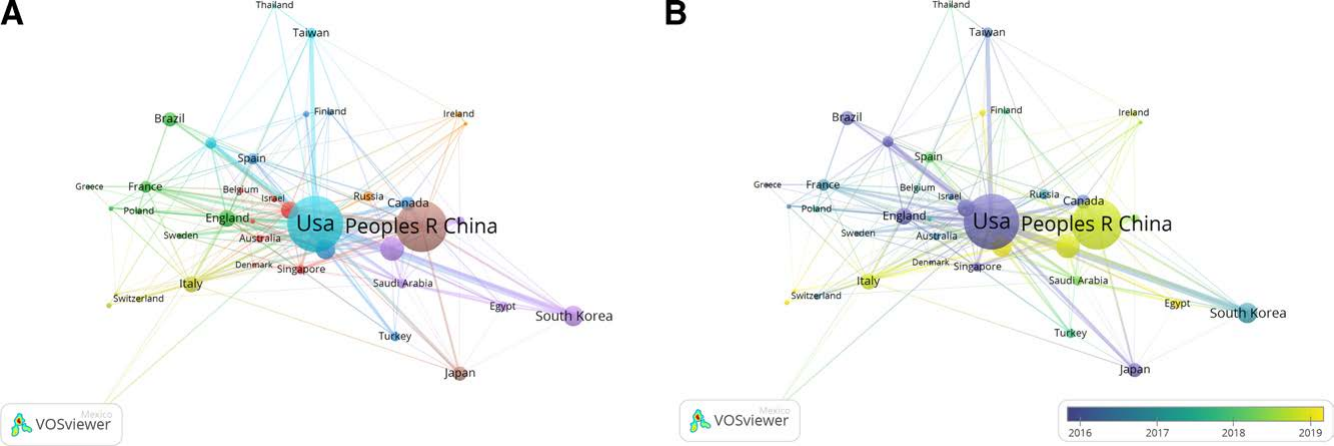
Co-authorship of countries. (A) Network map of country collaboration. (B) Overlay view of country collaboration based on time.

The top 10 contributing countries with the most of publications are distributed in Asia, North America, and Europe, primarily in Asia (5 countries). The top 5 countries are the USA (384 records), China (336 records), India (92 records), Korea (76 records), and Iran (71 records). The USA and China are also the 2 countries with the most total citations (USA:15,470 records; China: 8514 records), average article citations (USA: 51.70 records; China: 25.50 records), and the highest h-index (USA: 100; China: 72). The first article was published in 1992 by USA, while most of the other countries listed in Table 1 stepped into this field around 2000. The co-authorship based on the country and the time are shown in Figure 3A and B. The USA and China served as the 2 main investigation centers. The USA has close cooperation with Taiwan and the publication time is concentrated in 2015. China has close cooperation with Japan with more recent publications around 2019.

**Figure 4.**
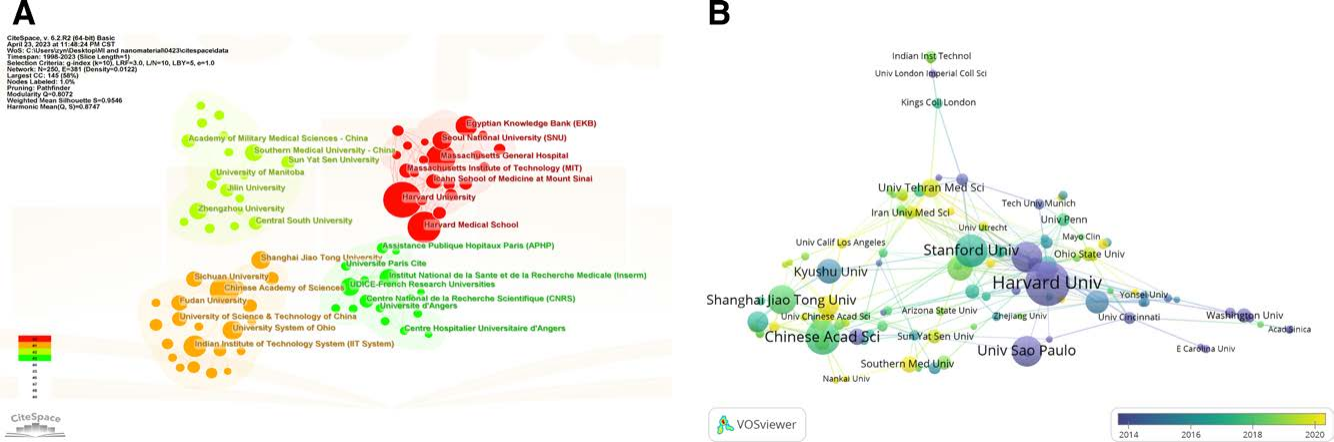
Cooperation of the top 4 clusters of institutions. (A) Network map of institution collaboration. (B) Timeline visualization map of institutions.

The contributions of institutions were also analyzed. Harvard University ranked first with 61 records and is followed by Harvard Medical School (47 records), the Chinese Academy of Sciences (36 records), Massachusetts General Hospital (35 records), and the University of California System (25 records). Notably, 6 institutions from the USA showed up in this top 10 list: Harvard University, Harvard Medical School, and Massachusetts General Hospital. The University of California System, the University of Texas System, Stanford University. Harvard University and Harvard Medical School also possessed comparably high total citations, average article citations, and h-index. Figure 4A showed the cooperation relationship between different institutions. Consistent with the previous results, the institutions’ cooperation showed a geographical distribution. Interestingly, there are 2 Chinese institution clusters. The University System of Ohio and the India Institute of Technology System cooperate with 1 Chinese institution cluster closely, including the Chinese Academy of Sciences, and Fudan University. The other Chinese institution cluster contains Jilin University, Zhengzhou University, and Central South University. The co-authorship analysis based on institutions and time was conducted as shown in Figure 4B. Chinese institutions were concentrated in recent years as represented by Shanghai Jiao Tong University, and Southern Medical University while Harvard University and Stanford University are relatively earlier regarding the publication time.

**Figure 5.**
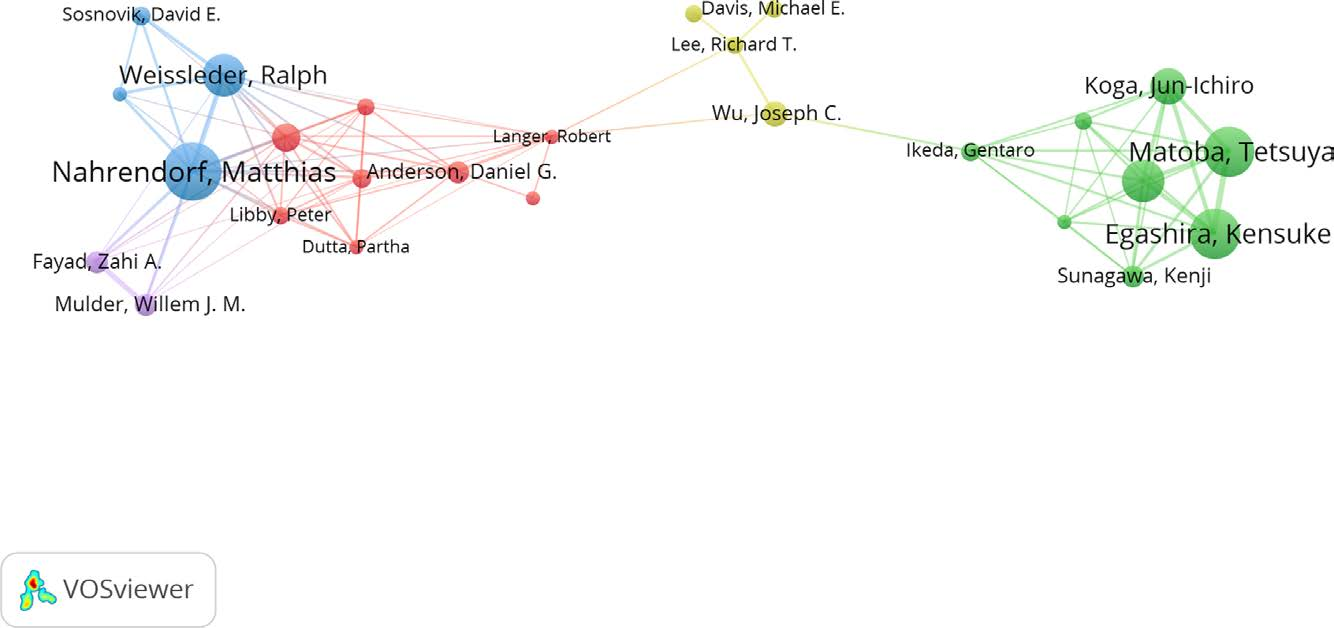
Cooperation of authors with a minimum number of 5 studies.

As for the contributing authors, Matthias Nahrendorf from the Center for Systems Biology, Massachusetts General Hospital is the top 1 in terms of publication records (23 records), total citations (3393 records) and h-index (22) (Table 1). The network map finally displayed the largest set of elements consisting of 26 authors (Fig. 5). There are 2 large clusters of nodes, which were connected by the author Joseph C. Wu, Richard T. Lee, and Michael E. Davis. Matthias Nahrendorf and Kensuke Egashira are 2 leading figures in these 2 clusters. We noticed active collaboration between Matthias Nahrendorf and Ralph Weissleder. The latter author ranked the second author on the list of top 10 authors by the h-index (Table 1). Kensuke Egashira and Tetsuya Matoba are also closely connected and are within the top 5 authors by the publications and h-index.

### 3.4. Co-citation analysis of author/reference/source

Co-cited references refer to references cited by other 2 publications at the same time and co-cited authors/journals were extracted and analyzed from the co-cited references (Table 2).

**Table 2:** The top 10 reference, authors, journals contributing to the area of smart nanomaterials biological application in myocardial infarction.

Rank	Top 10 references based on records		Top 10 authors based on records		Top 10 sources based on records		Top 10 sources based on h-index	
	Reference (publication time)	Record	Author	Records	Source	Records	Source	h-index
1	Katsuki S, 2014, CIRCULATION, V129, P896, DOI 10.1161/CIRCULATIONAHA.113.002870 (2014)	23	Egashira K	247	CIRCULATION	839	BIOSENSORS & BIOELECTRONICS	29
2	Ferreira MPA, 2017, SMALL, V13, P0, DOI 10.1002/smll.201701276 (2017)	21	Matoba T	247	J AM COLL CARDIOL	565	BIOMATERIALS	22
3	Kim K, 2016, BIOSENS BIOELECTRON, V77, P695, DOI 10.1016/j.bios.2015.10.008 (2016)	19	Nakano K	232	BIOMATERIALS	553	ANALYTICAL CHEMISTRY	14
4	Hao T, 2017, ACS NANO, V11, P5474, DOI 10.1021/acsnano.7b00221 (2017)	18	Nahrendorf M	176	P NATL ACAD SCI USA	550	INTERNATIONAL JOURNAL OF NANOMEDICINE	14
5	Bao R, 2017, BIOMATERIALS, V122, P63, DOI 10.1016/j.biomaterials.2017.01.012 (2017)	18	Sunagawa K	150	CIRCULATION RESEARCH	544	ACS NANO	13
6	Ahadian S, 2017, ACTA BIOMATER, V52, P81, DOI 10.1016/j.actbio.2016.12.009 (2016)	17	Weissleder R	146	NATURE	442	ACS APPLIED MATERIALS & INTERFACES	12
7	Jo H, 2017, TALANTA, V165, P442, DOI 10.1016/j.talanta.2016.12.091 (2016)	16	Koga J	129	ACS NANO	420	JOURNAL OF CONTROLLED RELEASE	12
8	Chang MY, 2013, J CONTROL RELEASE, V170, P287, DOI 10.1016/j.jconrel.2013.04.022 (2013)	16	Swirski Fk	113	NEW ENGL J MED	412	PLOS ONE	12
9	Shin SR, 2013, ACS NANO, V7, P2369, DOI 10.1021/nn305559j (2013)	16	Nakano Y	105	SCIENCE	402	SCIENTIFIC REPORTS	12
10	Yola ML, 2019, BIOSENS BIOELECTRON, V126, P418, DOI 10.1016/j.bios.2018.11.016 (2019)	16	Wang Cy	104	PLOS ONE	373	ACTA BIOMATERIALIA	11

**Figure 6.**
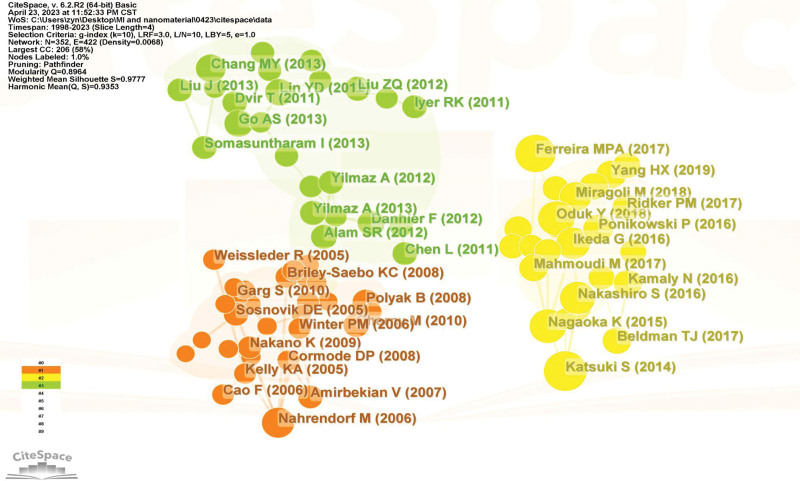
Network map of co-cited references with a minimum number of 30 citations.

The top 10 references based on records were led by the reference by Shunsuke Katsuki (record = 23). Of the 637,332 cited references, 15 met the threshold of 30 citations. Figure 6 showed the co-citation map of references. Notably, papers by Shunsuke Katsuki and Mónica P. A. Ferreira are clustered in the group of references, which were published mostly after 2014. The paper published in 2005 and 2006 by Matthias Nahrendorf and Ralph Weissleder are included in the orange cluster, which is full of publications before 2010 (Fig. 6).

**Figure 7.**
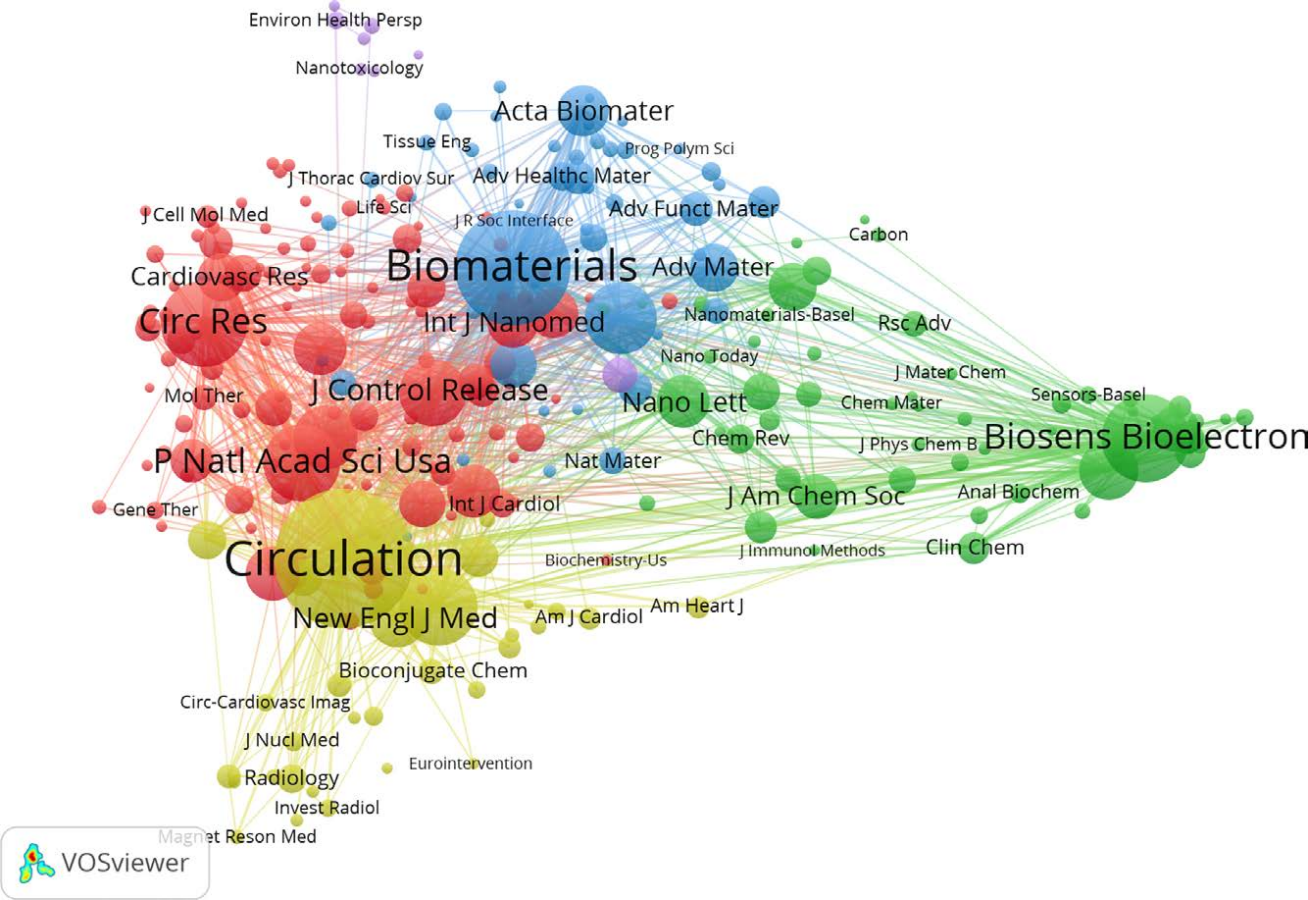
Network map of co-cited sources with a minimum number of 60 citations.

Of the 43,411 authors, 40 had at least 40 citations and the co-citation relationship of those are represented in Figure 7. Kensuke Egashira (record = 247), Tetsuya Matoba (record = 247), Kaku Nakano (record = 232), Matthias Nahrendorf (record = 176), and Kenji Sunagawa (record = 150) were the top 5 co-cited authors (Fig. 7).

**Figure 8.**
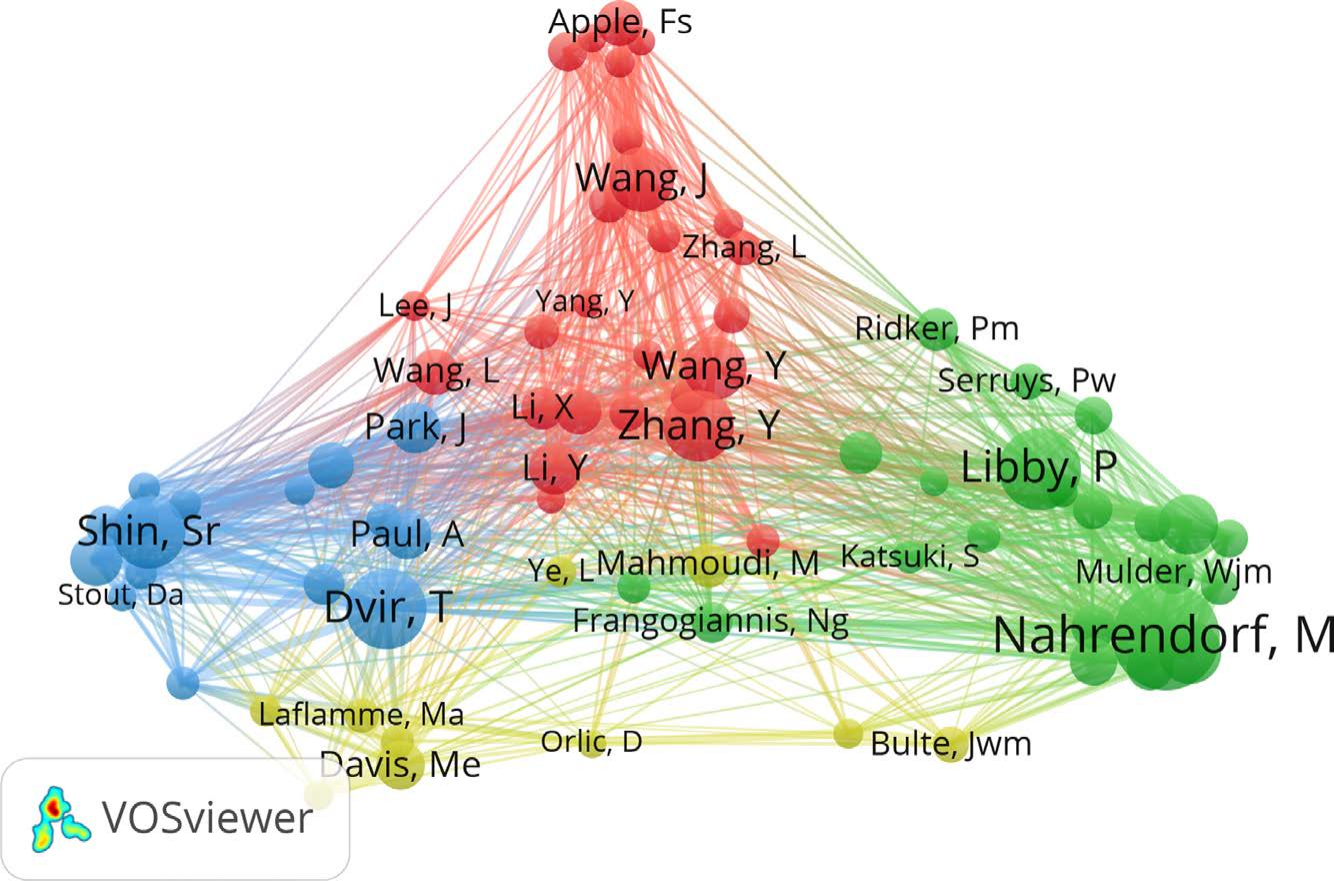
Network map of co-cited authors with a minimum number of 40 citations.

Of 7042 sources, 256 met the threshold of 60 citations and the co-citation map is shown in Figure 8. The top 5 journals with the highest citations are Circulation (record = 839), Journal of The American College of Cardiology (record = 565), and Biomaterials (record = 553). According to the top 10 journals with the highest publication impact, Biosensors & Bioelectronics, Biomaterials, and Analytical Chemistry are the top 3 journals with h-index of 29, 22, and 14, respectively.

### 3.5. Co-occurrence and burst analysis of keywords

**Figure 9.**
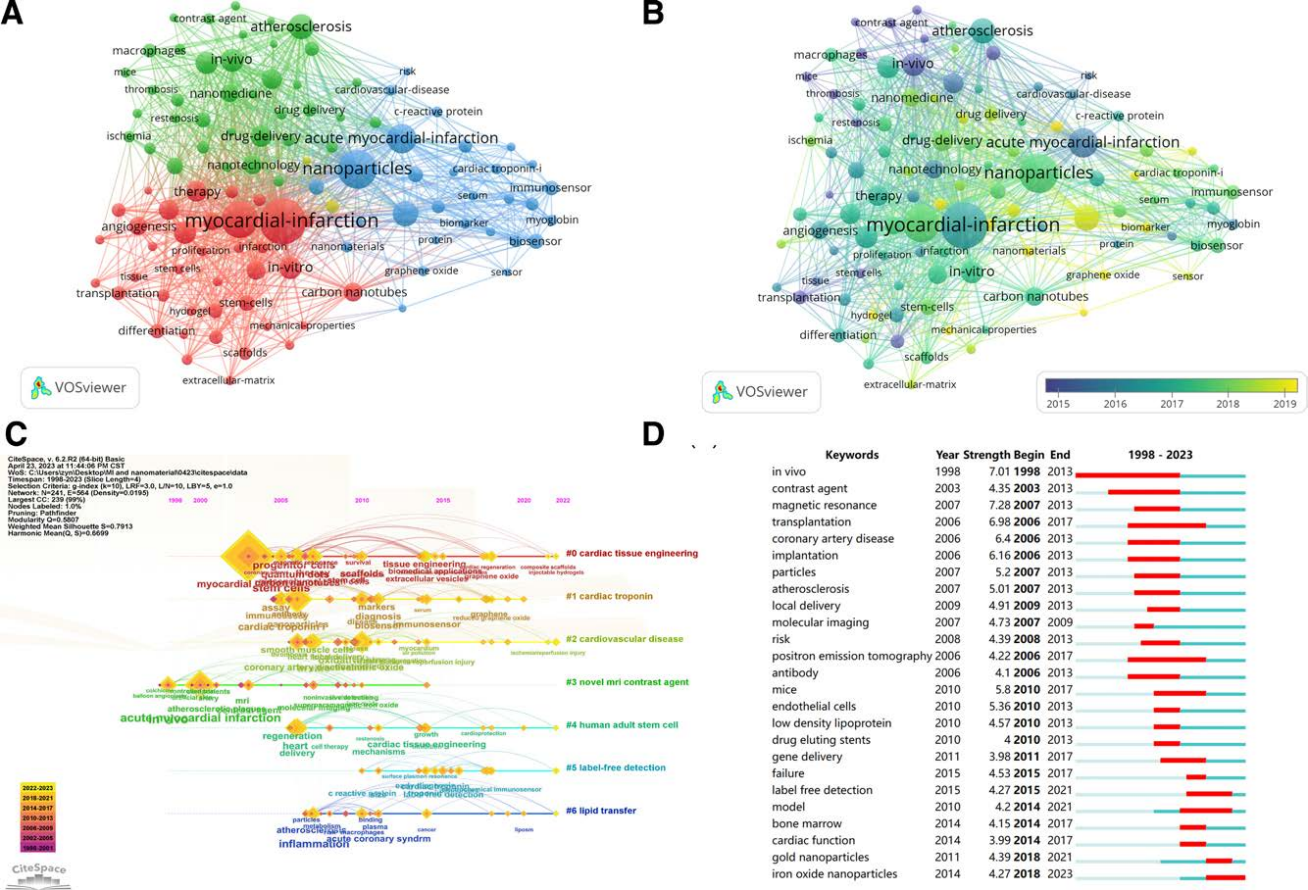
(A) Analysis of keywords. (A) Network map of the co-occurrence of keywords. (B) Overlay view of the co-occurrence of keywords based on time. (C) Timeline visualization map of keywords. (D) The top 25 keywords with the strongest citation burst in the last decade.

Through the keywords extracted from the titles and abstracts, we could capture the hot topics in a research field. Of 5808 keywords, 107 with a frequency of 20 and above were considered and analyzed in VOSviewer. Figure 9A showed the network map of the occurrence analysis. The 10 most common keywords are myocardial infarction (448 times), acute myocardial infarction (174 times), nanoparticles (138 times), drug delivery (121 times), gold nanoparticles (109 times), in vivo (99 times), delivery (93 times), in vitro (88 times), mesenchymal stem cells (88 times), and therapy (81 times). Figure 9B illustrated the evolution of keywords over time. Seven clusters were obtained from the analysis and applied in the timeline view by Citespace (Fig. 9C). The clusters with the most keywords are Cluster 0 represented by “cardiac tissue engineering,” including keywords like tissue engineering, progenitor cells, extracellular vesicles, etc. Cluster 1 is represented by “cardiac troponin,” including keywords like immunoassay, markers, graphene, biosensor, etc. Cluster 2 is represented by “cardiovascular disease,” including keywords like myocardium, smooth muscle cells, ischemia/reperfusion injury, etc. Cluster 3 is represented by “novel magnetic resonance imaging (MRI) contrast agent,” including keywords like MRI, and noninvasive. Cluster 4 is represented by “human adult stem cell,” including keywords like heart delivery, cardiac tissue engineering, and restenosis. Cluster 5 is represented by “label-free detection” including c-reactive protein, and surface plasmon resonance. Cluster 6 is represented by “lipid transfer” including inflammation, acute coronary syndrome, and liposm. Burst analysis of keywords can reveal the dynamic changes of frontiers over time. Figure 9D showed the top 25 burst words with a minimum duration of 2 years in the last decade. During the investigation of this field, scholars seemed to mainly focus on implantations, transplantations, and local delivery in the initial phase. In recent years, research paid more attention to the impact on endothelial cells, gene delivery, and cardiac functions. Gold nanoparticles and iron oxide nanoparticles appeared more frequently in the past 2 years indicating the present hotspots in this field.

## 4. Discussion

In our study, the top 10 countries with the most publications were displayed. Notably, the first 3 countries are USA, China, and India. Publications from USA and China also have the most total citations, average article citations, and the highest h-index. While the citations record and h-index value were relatively low in India, compared to other countries on the list. Regarding the top cited author and the authors with the most publications, results indicated that research teams led by Matthias Nahrendorf at Massachusetts General Hospital and Harvard Medical School and Egashira Kensuke at Kyushu University Graduate School of Medical Sciences are 2 major productive sources of vital publications in these fields, as the number of researchers from these groups accounts for more than half of the list.^[16–18]^ These results suggest a more collaborative and cooperative scientific research mode in the aforementioned groups and could be used as an excellent reference for other research groups. As for the top 5 co-cited references, 2 studies from China,^[19,20]^ 3 studies from Japan,^[18]^ Korea,^[21]^ and Finland^[22]^ respectively were highlighted, demonstrating scholars from Asia showed great potential in this scientific area. Among these 5 valuable articles, 4 focused on the therapeutic effect targeting different aspects of repairment after MI,^[18–20,22]^ and one focused on the detection and early diagnosis of MI.^[21]^

Interestingly, in Figure 5, the group of authors consisting of Joseph C. Wu, and Richard T. Lee connected the other 2 major research group clusters in USA and Asia. The paper published in 2017 in Nature Nanotechnology showed the cooperation between Joseph C. Wu, from Stanford Cardiovascular Institute, Stanford University School of Medicine, Richard T. Lee from the Department of Stem Cell and Regenerative Biology, Harvard University with Robert Langer from the David H. Koch Institute for Integrative Cancer Research, Massachusetts Institute of Technology.^[23]^ On the other hand, Joseph C. Wu, from Stanford Cardiovascular Institute, Stanford University School of Medicine also collaborated with Gentaro Ikeda from the same institute,^[24]^ who has abundant experience working with Egashira Kensuke, the leading research figure in the Department of Cardiovascular Medicine, Kyushu University Graduate School of Medical Sciences.^[25–29]^ The team of Gentaro Ikeda and Egashira Kensuke targeted ischemia–reperfusion injury and mitochondrial in myocardiocytes and has been productive since 2013.^[30,31]^ In 2014, they gave an abstract on poly(lactic acid/glycolic acid) (PLGA) nanoparticle-mediated targeting of a mitochondria division inhibitor, Mdivi-1, to protect the mitochondria from ischemia-reperfusion injury^[31]^ and started to investigate deeper in this area. Recently, they published a paper in the Journal of the American Heart Association about nanoparticle-mediated medicine that simultaneous targeting of mitochondrial injury and inflammation attenuates myocardial ischemia-reperfusion injury.^[28]^

Label-free detection, as the name suggests, is a technique that does not require the use of labels or probes to detect analytes of interest.^[32]^ Instead, it relies on the inherent properties of the molecules or cells being detected, such as their mass, size, or electrical charge.^[32,33]^ This approach has become increasingly popular in biomedical research, as it offers several advantages over traditional labeling techniques.^[33]^ One of the main advantages of label-free detection is that it can be performed in real time and with minimal sample preparation, making it a more efficient approach for high-throughput screening applications.^[34]^ Nanomaterials have emerged as a promising tool in label-free detection due to their unique optical, electrical, and magnetic properties. These materials can interact with analytes in a highly specific manner, enabling the detection of even low concentrations of target molecules. Some examples of nanomaterials commonly used in label-free detection include gold nanoparticles,^[34]^ carbon nanotubes,^[35]^ and quantum dots.^[36]^ One area where label-free detection and nano-materials have shown great potential is in the study of MI. Early detection of MI is crucial for effective treatment and improved patient outcomes, but current diagnostic methods often require invasive procedures or are limited in their sensitivity and specificity.^[37]^ Several studies have investigated the use of label-free detection and nanomaterials in MI research. For example, carbon nanotubes have been used to detect another MI biomarker, myoglobin, in blood samples.^[38]^ When myoglobin is present in a sample, it binds to the nanotubes and causes a change in their electrical conductivity, which can be detected and quantified.^[39]^ Label-free detection and nanomaterials offer exciting opportunities for research into MI diagnosis and provide new insights into the underlying mechanisms of MI.

Adult stem cells are multipotent cells that can differentiate into different cell types. The most common adult stem cell, mesenchymal stem cells (MSCs), was isolated from the bone marrow. The application of adult stem cells, particularly MSCs, is being investigated for treating various injuries or diseases like myocardial infarction,^[40]^ acute respiratory distress syndrome,^[41]^ acute kidney injury,^[42]^ and so on. In myocardial infarction, stem cell therapy aims to improve heart function by regenerating the damaged heart tissue. MSCs can differentiate into cardiomyocytes and contribute to tissue repair.^[43]^ In myocardial infarction research, nanomaterial-based platforms have been used to achieve various improvements.^[44]^ Nanobiomaterials have opened up new possibilities for incorporating cell therapy in curing MI by modulating cell functions. Studies have strived for synergistic effects when combining different characteristics of nanomaterials with cell therapy.^[44]^ The use of biocompatible nanomaterials is beneficial in delivering therapeutic molecules for angiogenesis and stem cell recruitment, which is crucial in the cardiac repair process.^[45]^ Moreover, nanomaterials possess unique properties such as nanotopography, electrical conductivity, and distinctive chemical characteristics which can modulate stem cells’ behaviors and greatly help the myocardiocyte repair process, especially in structural alignment, cardiac differentiation, and paracrine secretion aspects.^[46]^ Nonetheless, nanomaterials can also be employed to deliver therapeutic molecules such as growth factors together with the MSCs to enhance the treatment of myocardial infarction.^[47]^ MSCs are promising tools for regenerative medicine while nanomaterials provide new approaches to medical research. The combination of adult stem cells and nanomaterials has shown a great deal of promise in promoting post-MI tissue regeneration.

Although nanoparticles have demonstrated numerous capabilities that make them attractive for use in biology and medicine, the number of candidates that reach clinical trials or are commercialized is minimal compared with the constantly growing number of different formulations and extensive research around nanomaterials.^[48]^ This is primarily due to the “synthetic” and “foreign” nature of their surface, which can lead to strong off-target accumulation and adverse immune responses, caused by their recognition by immune phagocytes.^[49]^ As a result, only a small number of nanoparticles can develop their intended function. Cell-derived surfaces have become a popular alternative to artificial coatings or encapsulation methods by making use of natural cell membranes, in a way that mimics cell transfer.^[48,49]^ To date, cell membranes mimicking stem cell membranes,^[50]^ and macrophage membranes^[51]^ targeting treating cancers have been developed.^[50–52]^ In 2022, Bin He et al developed a biomimetic nanoparticle called thrombus-targeting and responsive biomimetic nanoparticle (PTPN) that targets thromboses and responds to the microenvironment for the treatment of MI. The PTPN contains a thrombus microenvironment-responsive phenylboronic acid nanocarrier, antioxidant molecule protocatechualdehyde, and tPA with thrombolytic effects, enclosed by a platelet membrane. The PTPN can adhere to damaged endothelial cells due to the platelet membrane’s thrombus-targeting capability. It disintegrates under slightly acidic conditions to reopen the ischemic artery. The released protocatechualdehyde eliminates reactive oxygen species induced by blood reperfusion and protects cardiomyocyte mitochondrial function from ischemic and reperfusion injury.^[53]^ Despite the potential safety concerns related to the use of natural components, the emergence of cell membrane-coated technology that replicates natural scenarios is expected to create a new category of nanocarriers that can be utilized for various biomedical purposes, and more papers are anticipated in the following years.

Gold nanoparticles have been gaining increasing attention in biomedical research due to their unique properties such as ease of synthesis, low toxicity, low immunogenicity, and stability.^[54]^ In the field of myocardial infarction, gold nanoparticles have demonstrated their potential as therapeutic and diagnostic agents. Gold nanoparticles can be functionalized with antibodies targeted to cardiac biomarkers such as troponin-1, which is a well-known cardiac enzyme because it is susceptible and specific to myocardial injuries.^[55]^ The binding of gold nanoparticles to these biomarkers allows for sensitive detection of troponin-1, providing a faster and more convenient diagnostic tool compared to traditional methods such as electrocardiogram and cardiac enzyme measurement.^[56]^ Moreover, gold nanoparticles can also be utilized as therapeutic agents in myocardial infarction.^[57]^ They can be functionalized with therapeutic agents such as statins, which exhibit anti-inflammatory and antioxidant properties.^[18,58]^ Further, the functionalization of gold nanoparticles with statins allows for targeted delivery to damaged cardiac tissue, resulting in more effective treatment with reduced side effects.^[59]^ The unique properties of gold nanoparticles make them an attractive option in the field of MI research. Their potential as both diagnostic and therapeutic agents offers exciting possibilities for improving the detection and treatment of this prevalent and potentially fatal condition.

One emerging field of therapeutic approaches to MI is the use of iron oxide nanoparticles (IONPs) for MI diagnosis, imaging, and therapy. IONPs have unique physiochemical properties, including their size, surface area, and magnetic behavior, which make them attractive and promising candidates for biomedical applications in MI.^[60]^ IONPs are frequently used as a contrast agent in cardiac MRI, allowing for early detection and accurate diagnosis of the disease.^[61]^ Similar to gold nanoparticles, IONPs could serve as a sensitive detector for myoglobin levels, which was widely recommended for early diagnosis of MI.^[38]^ Moreover, IONPs can be functionalized with drugs or mesenchymal stem cells, making them suitable for targeted drug delivery and therapy.^[62]^ This has the potential to improve the efficacy of current treatments and promote cardiac regeneration. While the use of IONPs in MI research shows great promise, some challenges need to be addressed.^[61,63]^ One major concern is the potential toxicity of IONPs to the heart and other organs. Studies have shown that the size and surface charge of IONPs can affect their toxicity, and further investigations are needed to determine the optimal IONP formulation for safe and effective MI therapy.^[64]^ Overall, the use of IONPs in MI research holds great potential to improve the diagnosis, treatment, and prognosis of this devastating disease. However, further research is needed to fully understand the benefits and limitations of IONPs in MI therapy and to develop safe and effective IONP formulations for clinical translation.

Our study has some limitations. Firstly, we only retrieved research papers from the database of WOS and only articles and reviews published in English were included, which might lead to incomplete data collection. Secondly, there is no date restriction in the database search process, which means some previously published papers might be missing the data that bibliography analysis needs. Additionally, we did not take the effect of publication time into consideration when using citation as an index of the contribution of articles. It is quite possible that some of the newly published impactful articles were not included in the top 10 reference list due to their short publication time.

## 5. Conclusions

To sum up, the biomedical applications of nanomaterials in MI have been a heated topic in recent years. The USA, China, and India are the leading countries and the most influential institution in the research is Harvard University. Circulation is the most-cited journal and Matoba, Tetsuya, and Egashira, Kensuke are the 2 most-cited authors. Worldwide collaboration is suggested to be strengthened. In clinical practice, MI remains a challenging situation, where more clinical trials and from-bench-to-bed basic research about nanomaterials biomedical applications are required.

## Author contributions

**Conceptualization:** Yi Chen, Jianna Zhang.

**Data curation:** Yi Chen.

**Formal analysis:** Jianna Zhang.

**Funding acquisition:** Jianna Zhang.

**Methodology:** Yi Chen, Jianna Zhang.

**Software:** Yi Chen.

**Supervision:** Jianna Zhang.

**Validation:** Yi Chen.

**Visualization:** Jianna Zhang.

**Writing – original draft:** Yi Chen.

**Writing – review & editing:** Jianna Zhang.

## Supplementary Material


