# Supplementary material for: A bibliography of smart nanomaterials biological application in myocardial infarction research
Source: Medicine (Baltimore). 2024 Apr 5;103(14):e37672. doi: 10.1097/MD.0000000000037672 (PMC10994481; doi:10.1097/MD.0000000000037672)
Supplement: Supplementary file 1 [file medi-103-e37672-s001.docx]

Title: A Bibliography of Smart Nanomaterials Biological Application in Myocardial Infarction Research

First author: Yi Chen, M.D.

Search terms:

#1 TS=((nanocomposite) OR (nanosheet) OR (nanorod) OR (nanotube) OR (nanofiber) OR (nanosphere) OR (nanomaterial) OR (nanodot) OR (nanomedicine) OR (nanotechnology) OR (nanocrystal) OR (nanocrystalline material) OR (nano particle) OR (nanoparticle)

OR (Hydrogel) or (Hydrogel, Patterned) or (Patterned Hydrogel) or (Patterned Hydrogels) or (Hydrogel, In Situ) or (In Situ Hydrogel) or (In Situ Hydrogels))

#2 TS=((myocardial infarction) or (acute coronary syndrome) or (cardiac infarction) or (coronary artery disease) or (heart attack) or (heart infarction))

#1 AND #2
